# Bis(3-amino­pyrazine-2-carboxyl­ato-κ^2^ *N* ^1^,*O*)di­aqua­nickel(II) dihydrate

**DOI:** 10.1107/S1600536813012208

**Published:** 2013-05-11

**Authors:** Rafika Bouchene, Amina Khadri, Sofiane Bouacida, Fadila Berrah, Hocine Merazig

**Affiliations:** a Laboratoire de Chimie Appliquée et Technologie des Matériaux LCATM, Université Oum El Bouaghi, Algeria; b Département Sciences de la Matière, Faculté des Sciences Exactes et Sciences de la Nature et de la Vie, Université Oum El Bouaghi, Algeria; c Unité de Recherche de Chimie de l’Environnement et Moléculaire Structurale, CHEMS, Faculté des Sciences Exactes, Université Constantine 25000, Algeria

## Abstract

In the title compound, [Ni(C_5_H_4_N_3_O_2_)_2_(H_2_O)_2_]·2H_2_O, the Ni^II^ ion lies on an inversion center and is coordinated in an slightly distorted octa­hedral environment by two N,O-chelating 3-amino­pyrazine-2-carboxyl­ate (APZC) ligands in the equatorial plane and two *trans*-axial aqua ligands. In the crystal, O—H⋯O, N—H⋯O and O—H⋯N hydrogen bonds involving the solvent water mol­ecules, aqua and APZC ligands form layers parallel to (010). These layers are linked further *via* O—H⋯O, N—H⋯O and C—H⋯O hydrogen bonds involving the axial aqua ligands, amino groups and the carboxyl­ate groups of the APZC ligands, forming a three-dimensional network.

## Related literature

For background to hybrid compounds, see: Bouchene *et al.* (2013 ▶); Bouacida *et al.* (2007 ▶, 2009 ▶). For the structure of the non-hydrated analogue, see: Ptasiewicz-Bak & Leciejewicz (1999 ▶). For 3-amino­pyrazine-2-carboxyl­ate–metal (*M*) complexes, see: Bouchene *et al.* (2013 ▶) [*M* = Co(II)]; Leciejewicz *et al.* (1997 ▶) [*M* = Ca(II)]; Leciejewicz *et al.* (1998 ▶) [*M* = Sr(II)]; Ptasiewicz-Bak & Leciejewicz (1997 ▶) [*M* = Mg(II)]; Tayebee *et al.* (2008 ▶) [*M* = Na(I)]; Ptasiewicz-Bak & Leciejewicz (1999 ▶). For proprieties and applications of pyrazine-2-carb­oxy­lic acid, see: Zhang & Mitchison (2003 ▶); Manju & Chaudhary, (2010 ▶); Chanda & Sangeetika (2004 ▶).
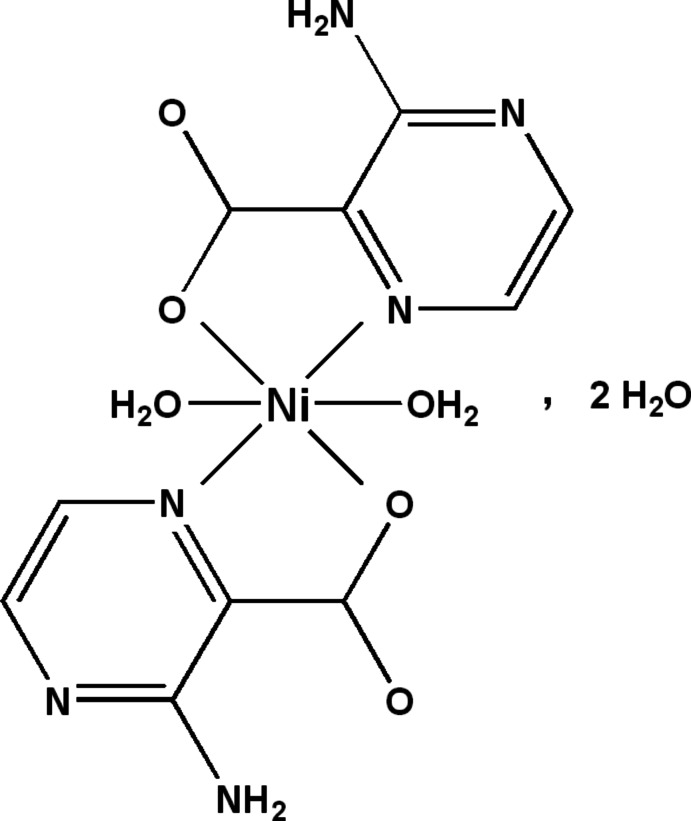


## Experimental

### 

#### Crystal data

[Ni(C_5_H_4_N_3_O_2_)_2_(H_2_O)_2_]·2H_2_O
*M* *_r_* = 406.98
Monoclinic, 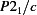

*a* = 9.7939 (15) Å
*b* = 5.1123 (9) Å
*c* = 16.776 (3) Å
β = 115.838 (11)°
*V* = 756.0 (2) Å^3^
*Z* = 2
Mo *K*α radiation
μ = 1.34 mm^−1^
*T* = 150 K
0.18 × 0.16 × 0.15 mm

#### Data collection

Bruker APEXII CCD diffractometer
6200 measured reflections
1326 independent reflections
1121 reflections with *I* > 2σ(*I*)
*R*_int_ = 0.051

#### Refinement

*R*[*F* ^2^ > 2σ(*F* ^2^)] = 0.028
*wR*(*F* ^2^) = 0.073
*S* = 1.04
1326 reflections
127 parameters
H atoms treated by a mixture of independent and constrained refinement
Δρ_max_ = 0.36 e Å^−3^
Δρ_min_ = −0.59 e Å^−3^

### 

Data collection: *APEX2* (Bruker, 2011 ▶); cell refinement: *SAINT* (Bruker, 2011 ▶); data reduction: *SAINT*; program(s) used to solve structure: *SIR2002* (Burla *et al.*, 2005 ▶); program(s) used to refine structure: *SHELXL97* (Sheldrick, 2008 ▶); molecular graphics: *ORTEP-3 for Windows* (Farrugia, 2012 ▶) and *DIAMOND* (Brandenburg & Berndt, 2001 ▶); software used to prepare material for publication: *WinGX* (Farrugia, 2012 ▶).

## Supplementary Material

Crystal structure: contains datablock(s) global, I. DOI: 10.1107/S1600536813012208/lh5610sup1.cif

Structure factors: contains datablock(s) I. DOI: 10.1107/S1600536813012208/lh5610Isup2.hkl

Additional supplementary materials:  crystallographic information; 3D view; checkCIF report

## Figures and Tables

**Table 1:** Hydrogen-bond geometry (Å, °)

*D*—H⋯*A*	*D*—H	H⋯*A*	*D*⋯*A*	*D*—H⋯*A*
O1*W*—H1*A*⋯O2*W* ^i^	0.83 (3)	1.97 (3)	2.789 (3)	169 (3)
O1*W*—H1*B*⋯O1^ii^	0.75 (3)	1.94 (3)	2.690 (3)	176 (4)
N3—H1*N*⋯O2*W* ^iii^	0.86	2.27	3.117 (3)	168
O2*W*—H2*A*⋯O2*W* ^iv^	0.77 (3)	2.12 (3)	2.867 (3)	164 (4)
O2*W*—H2*B*⋯N2^v^	0.78 (3)	2.03 (3)	2.792 (3)	168 (3)
N3—H2*N*⋯O2	0.86	2.10	2.733 (3)	130
N3—H2*N*⋯O2^vi^	0.86	2.20	2.871 (3)	135
C5—H5⋯O1*W* ^vii^	0.93	2.54	3.377 (4)	150

Symmetry codes: (i) 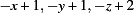; (ii) 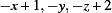; (iii) 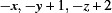; (iv) 
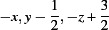
; (v) 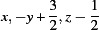; (vi) 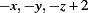; (vii) 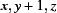.

**Fig. 1.**
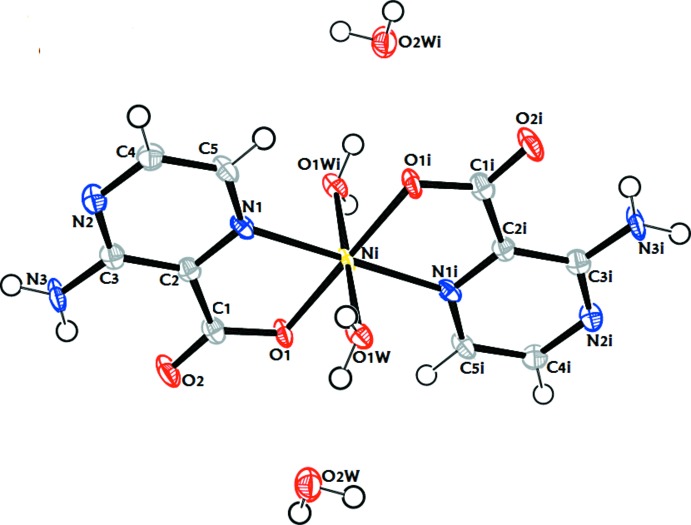
The molecular structure of the title compound. Displacement ellipsoids are drawn at the 50% probability level. H atoms are represented as small spheres of arbitrary radii. Symmetry code: (i)-x+1, -y+1, -z+2.

**Fig. 2.**
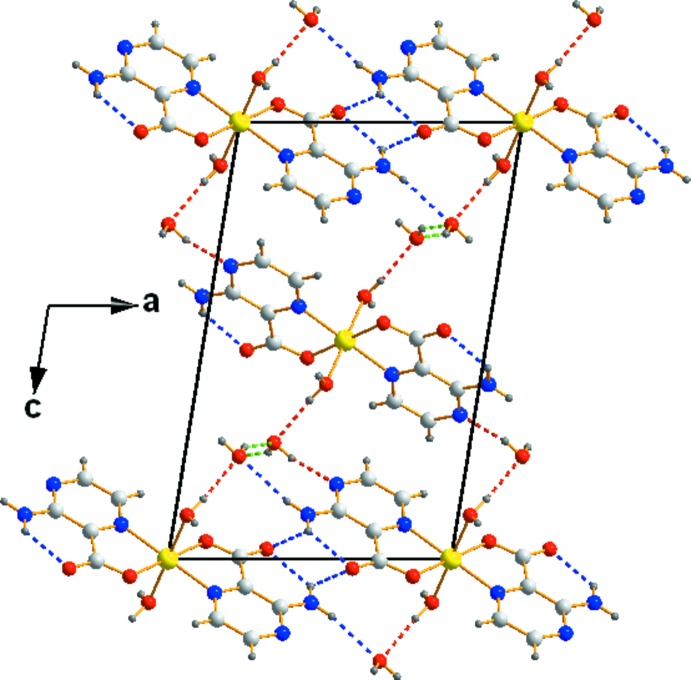
Partial packing plot viewed along the b axis showing the hydrogen bonds (dashed lines) forming layers.

**Fig. 3.**
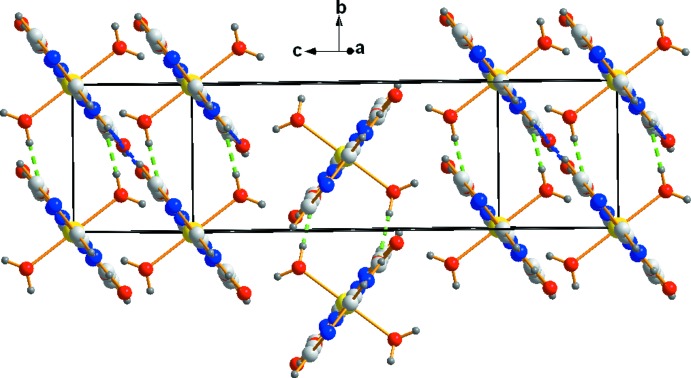
Partial packing of (I) showing only the O—H···O hydrogen bonds which connect the layers in Fig. 2 into a three-dimensional network.

## supplementary crystallographic information

### Comment

The pyrazine-2-carboxylic acid bridging ligand, owing to its ability to act in acidic environments (Zhang & Mitchison, 2003), has been extensively studied for biological applications, such as anti-tubercular (Manju *et al.*, 2010), antipyretic, antitumor, and anticancer (Chanda *et al.*, 2004). An additional amino substitution on 3-amino-2-pyrazine carboxylic acid could be expected to enhance crystal packing through extensive hydrogen bonding. APZC has a large variety of coordination geometries in metal complexes (Leciejewicz *et al.*, 1997, 1998; Ptasiewicz-Bak & Leciejewicz, 1997; Tayebee *et al.*, 2008).

In continuation of our search to enrich the variety of such kinds of hybrid compounds and to investigate the influence of hydrogen bonds on the structural features (Bouacida *et al.*, 2007, 2009), we report here the synthesis and crystal structure of the title compound, (I), as a extention of our earlier work on N,O chelated ligands (Bouchene *et al.*2013) which can be involved in covalent interactions in metal coordination chemistry.

The asymmetric unit of (I) consists of one-half of the molecule, with the other half generated by a crystallographic inversion center. The molecular structure is shown in Fig. 1. The Ni^II^ ion is coordinated by two 3-amino-2-pyrazine carboxylate ligands *via* N,O-chelating groups in the equatorial plane and two aqua O atoms in the axial sites forming a slightly distorted octahedral coordination environment. The Ni—N, Ni—O and Ni—O_aqua_ distances are consistent with the reported data for the anhydrous Ni(II)(APZC)_2_(H_2_O)_2_ complex (Ptasiewicz-Bak & Leciejewicz, 1999). In the crystal, the solvent water molecules and complex molecules are involved in intermolecular O—H···O, O—H···N and N—H···O hydrogen bonds forming two-dimensional layers parallel to (010) (Fig.2). Further O—H···O hydrogen bonds (Fig.3) involving the aqua ligands, N—H···.O hydrogen bonds the carboxylate groups of the APZC ligands form a three-dimensional network.

### Experimental

Nickel dichloride hexahydrate (0.2 mmol) and 3-aminopyrazine-2-carboxylic acid (0.02 mmol) were dissolved in acidified water with concentrated hydrogen chloride acid (37%). Light green crystals, suitable for X-ray diffraction study, were obtained from evaporation of obtained solution for three days.

### Refinement

The H atoms bonded to C and N were located in differnce Fourier maps but subsequently introduced in calculated positions and treated as riding on their parent atoms (C or N) with C—H = 0.93 Å and N—H = 0.86 Å with *U*_iso_(H) = 1.2*U*_eq_(C or N). Atoms H1W and H2W were located in a difference Fourier map and refined isotropically wirh *U*_iso_(H) = 1.5*U*_eq_(O).

### Crystal data

**Table:**

[Ni(C_5_H_4_N_3_O_2_)_2_(H_2_O)_2_]·2H_2_O	*F*(000) = 420
*M**_r_* = 406.98	*D*_x_ = 1.788 Mg m^−^^3^
Monoclinic, *P*2_1_/*c*	Mo *K*α radiation, λ = 0.71073 Å
*a* = 9.7939 (15) Å	Cell parameters from 2285 reflections
*b* = 5.1123 (9) Å	θ = 2.7–25°
*c* = 16.776 (3) Å	µ = 1.34 mm^−^^1^
β = 115.838 (11)°	*T* = 150 K
*V* = 756.0 (2) Å^3^	Cube, white
*Z* = 2	0.18 × 0.16 × 0.15 mm

### Data collection

**Table:**

Bruker APEXII CCD diffractometer	1121 reflections with *I* > 2σ(*I*)
Radiation source: sealed tube	*R*_int_ = 0.051
Graphite monochromator	θ_max_ = 25.1°, θ_min_ = 2.7°
φ and ω scans	*h* = −11→11
6200 measured reflections	*k* = −6→6
1326 independent reflections	*l* = −19→19

### Refinement

**Table:**

Refinement on *F*^2^	Primary atom site location: structure-invariant direct methods
Least-squares matrix: full	Secondary atom site location: difference Fourier map
*R*[*F*^2^ > 2σ(*F*^2^)] = 0.028	Hydrogen site location: inferred from neighbouring sites
*wR*(*F*^2^) = 0.073	H atoms treated by a mixture of independent and constrained refinement
*S* = 1.04	*w* = 1/[σ^2^(*F*_o_^2^) + (0.0363*P*)^2^ + 0.4886*P*] where *P* = (*F*_o_^2^ + 2*F*_c_^2^)/3
1326 reflections	(Δ/σ)_max_ < 0.001
127 parameters	Δρ_max_ = 0.36 e Å^−^^3^
0 restraints	Δρ_min_ = −0.59 e Å^−^^3^

### Special details

**Table:**

Geometry. All e.s.d.'s (except the e.s.d. in the dihedral angle between two l.s. planes) are estimated using the full covariance matrix. The cell e.s.d.'s are taken into account individually in the estimation of e.s.d.'s in distances, angles and torsion angles; correlations between e.s.d.'s in cell parameters are only used when they are defined by crystal symmetry. An approximate (isotropic) treatment of cell e.s.d.'s is used for estimating e.s.d.'s involving l.s. planes.
Refinement. Refinement of *F*^2^ against ALL reflections. The weighted *R*-factor *wR* and goodness of fit *S* are based on *F*^2^, conventional *R*-factors *R* are based on *F*, with *F* set to zero for negative *F*^2^. The threshold expression of *F*^2^ > σ(*F*^2^) is used only for calculating *R*-factors(gt) *etc*. and is not relevant to the choice of reflections for refinement. *R*-factors based on *F*^2^ are statistically about twice as large as those based on *F*, and *R*- factors based on ALL data will be even larger.

### Fractional atomic coordinates and isotropic or equivalent isotropic displacement parameters (Å^2^)

**Table:**

	*x*	*y*	*z*	*U*_iso_*/*U*_eq_	
C1	0.2428 (3)	0.2367 (5)	0.99519 (16)	0.0089 (5)	
C2	0.2750 (3)	0.4494 (4)	1.06347 (15)	0.0079 (5)	
C3	0.1864 (3)	0.4915 (5)	1.11094 (15)	0.0090 (5)	
C4	0.3430 (3)	0.8353 (5)	1.18295 (16)	0.0098 (5)	
H4	0.3692	0.9711	1.2239	0.012*	
C5	0.4304 (3)	0.7942 (5)	1.13805 (15)	0.0097 (5)	
H5	0.5141	0.8999	1.1492	0.012*	
N1	0.3937 (2)	0.6010 (4)	1.07831 (13)	0.0074 (4)	
N2	0.2227 (2)	0.6890 (4)	1.17019 (13)	0.0102 (4)	
N3	0.0696 (2)	0.3396 (4)	1.10092 (14)	0.0125 (5)	
H1N	0.0202	0.3689	1.1316	0.015*	
H2N	0.0436	0.2122	1.0637	0.015*	
O1	0.33555 (17)	0.2240 (3)	0.96010 (11)	0.0090 (4)	
O2	0.13487 (18)	0.0885 (3)	0.97784 (12)	0.0137 (4)	
O1W	0.6426 (2)	0.2542 (4)	1.10017 (12)	0.0102 (4)	
H1A	0.726 (3)	0.312 (6)	1.1356 (19)	0.015*	
H1B	0.652 (3)	0.124 (6)	1.083 (2)	0.015*	
O2W	0.0735 (2)	0.6137 (4)	0.76616 (12)	0.0133 (4)	
H2A	0.046 (4)	0.472 (6)	0.754 (2)	0.02*	
H2B	0.104 (3)	0.663 (6)	0.733 (2)	0.02*	
Ni1	0.5	0.5	1	0.00664 (16)	

### Atomic displacement parameters (Å^2^)

**Table:**

	*U*^11^	*U*^22^	*U*^33^	*U*^12^	*U*^13^	*U*^23^
C1	0.0073 (11)	0.0071 (12)	0.0116 (12)	0.0010 (9)	0.0036 (10)	0.0014 (10)
C2	0.0067 (12)	0.0078 (12)	0.0087 (11)	0.0002 (9)	0.0029 (10)	0.0009 (9)
C3	0.0078 (11)	0.0088 (12)	0.0098 (11)	0.0029 (10)	0.0031 (9)	0.0029 (11)
C4	0.0114 (12)	0.0077 (12)	0.0105 (12)	−0.0014 (9)	0.0050 (10)	−0.0024 (10)
C5	0.0068 (12)	0.0089 (12)	0.0137 (13)	−0.0011 (9)	0.0048 (10)	−0.0017 (10)
N1	0.0040 (10)	0.0067 (10)	0.0102 (10)	0.0004 (8)	0.0019 (8)	0.0009 (8)
N2	0.0097 (10)	0.0101 (11)	0.0123 (10)	−0.0008 (8)	0.0061 (9)	0.0001 (8)
N3	0.0094 (10)	0.0139 (11)	0.0195 (11)	−0.0052 (9)	0.0113 (9)	−0.0056 (9)
O1	0.0069 (8)	0.0093 (8)	0.0137 (9)	−0.0018 (7)	0.0073 (7)	−0.0032 (7)
O2	0.0092 (9)	0.0135 (9)	0.0213 (10)	−0.0055 (7)	0.0092 (8)	−0.0059 (8)
O1W	0.0077 (9)	0.0081 (9)	0.0147 (9)	−0.0015 (7)	0.0046 (8)	−0.0030 (8)
O2W	0.0148 (10)	0.0129 (9)	0.0169 (10)	−0.0035 (8)	0.0115 (8)	−0.0018 (8)
Ni1	0.0049 (2)	0.0062 (2)	0.0106 (2)	−0.00089 (17)	0.00506 (18)	−0.00108 (18)

### Geometric parameters (Å, º)

**Table:**

C1—O2	1.228 (3)	N1—Ni1	2.0657 (19)
C1—O1	1.281 (3)	N3—H1N	0.86
C1—C2	1.510 (3)	N3—H2N	0.86
C2—N1	1.327 (3)	O1—Ni1	2.0233 (16)
C2—C3	1.427 (3)	O1W—Ni1	2.0755 (18)
C3—N3	1.331 (3)	O1W—H1A	0.83 (3)
C3—N2	1.351 (3)	O1W—H1B	0.74 (3)
C4—N2	1.332 (3)	O2W—H2A	0.77 (3)
C4—C5	1.381 (3)	O2W—H2B	0.78 (3)
C4—H4	0.93	Ni1—O1^i^	2.0233 (16)
C5—N1	1.340 (3)	Ni1—N1^i^	2.066 (2)
C5—H5	0.93	Ni1—O1W^i^	2.0755 (18)
			
O2—C1—O1	124.7 (2)	H1N—N3—H2N	120
O2—C1—C2	119.8 (2)	C1—O1—Ni1	115.72 (15)
O1—C1—C2	115.5 (2)	Ni1—O1W—H1A	118 (2)
N1—C2—C3	120.2 (2)	Ni1—O1W—H1B	113 (2)
N1—C2—C1	116.0 (2)	H1A—O1W—H1B	110 (3)
C3—C2—C1	123.7 (2)	H2A—O2W—H2B	108 (3)
N3—C3—N2	117.7 (2)	O1—Ni1—O1^i^	180
N3—C3—C2	122.7 (2)	O1—Ni1—N1	80.54 (7)
N2—C3—C2	119.6 (2)	O1^i^—Ni1—N1	99.46 (7)
N2—C4—C5	122.8 (2)	O1—Ni1—N1^i^	99.46 (7)
N2—C4—H4	118.6	O1^i^—Ni1—N1^i^	80.54 (7)
C5—C4—H4	118.6	N1—Ni1—N1^i^	180.0000 (10)
N1—C5—C4	119.5 (2)	O1—Ni1—O1W^i^	89.81 (7)
N1—C5—H5	120.3	O1^i^—Ni1—O1W^i^	90.19 (7)
C4—C5—H5	120.3	N1—Ni1—O1W^i^	90.92 (7)
C2—N1—C5	119.9 (2)	N1^i^—Ni1—O1W^i^	89.08 (7)
C2—N1—Ni1	112.20 (15)	O1—Ni1—O1W	90.19 (7)
C5—N1—Ni1	127.92 (16)	O1^i^—Ni1—O1W	89.81 (7)
C4—N2—C3	117.9 (2)	N1—Ni1—O1W	89.08 (7)
C3—N3—H1N	120	N1^i^—Ni1—O1W	90.92 (7)
C3—N3—H2N	120	O1W^i^—Ni1—O1W	180.00 (9)
			
O2—C1—C2—N1	180.0 (2)	N3—C3—N2—C4	177.5 (2)
O1—C1—C2—N1	1.0 (3)	C2—C3—N2—C4	−1.1 (3)
O2—C1—C2—C3	0.2 (4)	O2—C1—O1—Ni1	178.74 (18)
O1—C1—C2—C3	−178.8 (2)	C2—C1—O1—Ni1	−2.3 (3)
N1—C2—C3—N3	−177.5 (2)	C1—O1—Ni1—N1	2.15 (16)
C1—C2—C3—N3	2.3 (4)	C1—O1—Ni1—N1^i^	−177.85 (16)
N1—C2—C3—N2	1.0 (3)	C1—O1—Ni1—O1W^i^	−88.81 (16)
C1—C2—C3—N2	−179.2 (2)	C1—O1—Ni1—O1W	91.19 (16)
N2—C4—C5—N1	0.5 (4)	C2—N1—Ni1—O1	−1.50 (15)
C3—C2—N1—C5	0.0 (3)	C5—N1—Ni1—O1	179.2 (2)
C1—C2—N1—C5	−179.9 (2)	C2—N1—Ni1—O1^i^	178.50 (15)
C3—C2—N1—Ni1	−179.41 (17)	C5—N1—Ni1—O1^i^	−0.8 (2)
C1—C2—N1—Ni1	0.8 (2)	C2—N1—Ni1—O1W^i^	88.16 (16)
C4—C5—N1—C2	−0.7 (3)	C5—N1—Ni1—O1W^i^	−91.1 (2)
C4—C5—N1—Ni1	178.57 (17)	C2—N1—Ni1—O1W	−91.84 (16)
C5—C4—N2—C3	0.4 (3)	C5—N1—Ni1—O1W	88.9 (2)

Symmetry code: (i) −*x*+1, −*y*+1, −*z*+2.

### Hydrogen-bond geometry (Å, º)

**Table:**

*D*—H···*A*	*D*—H	H···*A*	*D*···*A*	*D*—H···*A*
O1*W*—H1*A*···O2*W*^i^	0.83 (3)	1.97 (3)	2.789 (3)	169 (3)
O1*W*—H1*B*···O1^ii^	0.75 (3)	1.94 (3)	2.690 (3)	176 (4)
N3—H1*N*···O2*W*^iii^	0.86	2.27	3.117 (3)	168
O2*W*—H2*A*···O2*W*^iv^	0.77 (3)	2.12 (3)	2.867 (3)	164 (4)
O2*W*—H2*B*···N2^v^	0.78 (3)	2.03 (3)	2.792 (3)	168 (3)
N3—H2*N*···O2	0.86	2.10	2.733 (3)	130
N3—H2*N*···O2^vi^	0.86	2.20	2.871 (3)	135
C5—H5···O1*W*^vii^	0.93	2.54	3.377 (4)	150

Symmetry codes: (i) −*x*+1, −*y*+1, −*z*+2; (ii) −*x*+1, −*y*, −*z*+2; (iii) −*x*, −*y*+1, −*z*+2; (iv) −*x*, *y*−1/2, −*z*+3/2; (v) *x*, −*y*+3/2, *z*−1/2; (vi) −*x*, −*y*, −*z*+2; (vii) *x*, *y*+1, *z*.
